# Tissue engineering a tendon-bone junction with biodegradable braided scaffolds

**DOI:** 10.1186/s40824-019-0160-3

**Published:** 2019-05-16

**Authors:** Harshini Ramakrishna, Tieshi Li, Ting He, Joseph Temple, Martin W. King, Anna Spagnoli

**Affiliations:** 1 Wilson College of Textiles, North Carolina State University, 1020 Main Campus Drive, Raleigh, NC 27606 USA; 2 Department of Pediatrics, Rush University Medical Center, 1735 W. Harrison Street, 502A Cohn Research Building, 5th floor, Chicago, IL 60612 USA; 3 College of Textiles, Donghua University, 2999 Renmin Road North, Songjiang District, Shanghai, 201620 China; 4 Department of Biosystems Engineering, University of Manitoba, Engineering, Information and Technology Complex, 75A Chancellor’s Circle, Winnipeg, MB R3T 5V6 Canada; 5 Department of Pediatrics, University of Nebraska Medical Center, Children’s Hospital & Medical Center, Omaha, NE 68198-5945 USA

**Keywords:** Tendon bone junction, Polylactic acid, Biodegradable, Braided scaffold, Tgfbr2 expressing joint progenitor cells

## Abstract

**Background:**

Tendons play an important role in transferring stress between muscles and bones and in maintaining the stability of joints. Tendon tears are difficult to heal and are associated with high recurrence rates. So, the objective of this study was to develop a biodegradable scaffold for tendon-bone junction regeneration.

**Methods:**

Two types of polylactic acid (PLA) yarns, having fibers with round and four deep grooved cross-sections, were braided into tubular scaffolds and cultured with murine Transforming growth factor beta type II receptor (Tgfbr2)-expressing joint progenitor cells. The scaffolds were designed to mimic the mechanical, immuno-chemical and biological properties of natural mouse tendon-bone junctions. Three different tubular scaffolds measuring 2 mm in diameter were braided on a Steeger 16-spindle braiding machine and biological and mechanical performance of the three scaffolds were evaluated.

**Results:**

The mechanical test results indicated that three different braided scaffold structures provided a wide range of mechanical properties that mimic the components of tendon bone junction and results of the biological tests confirmed cell viability, active cell attachment and proliferation throughout all three scaffolds.

**Conclusions:**

This study has identified that the three proposed types of braided scaffolds with some improvement in their structures have the potential to be used as scaffolds for the regeneration of a tendon bone tissue junction.

## Background

The tendon-bone junction is a functionally graded tissue material, which provides the transition from a flexible and soft tissue tendon to hard mineralized bone. It also plays an important role in transferring mechanical stresses between muscles and bones and in maintaining the stability of joints. Tendon tears have a poor healing capacity, and the most common tendon bone junction injuries are at the Achilles tendon, the rotator cuff and the anterior cruciate ligament. Every year there are about 100,000 ACL reconstructive surgeries, 75,000 rotator cuff repairs and 230,000 Achilles tendon repairs performed in the United States [1, 2].

In general, torn and injured tendons can be restored successfully by appropriate surgery, but the functionally graded transitional zone at the tendon-bone interface is not regenerated. Thus, one of the most immediate challenges facing the field of regenerative medicine is “Interfacial Tissue Engineering” (ITE), which addresses the question of how to generate a multiple tissue junction such as a tendon-bone interface which has integrity, continuity and consists of at least two different yet contiguous types of cells, including tenocytes and osteoblasts [1].

Research to date has taken the approach that it is necessary to use pluripotent stem cells or to co-culture the two dissimilar cell lines either sequentially or together in a single compromised media and under co-culture conditions [1, 3–5]. This simplistic approach assumes that a tissue junction consists of only two types of cells that join at the interface.

Many previous developmental studies on mouse embryos have shown evidence of a distinct intermediate interfacial tissue type between the bone and the tendon [6]. Our approach has been to focus on the unique joint TGF-β-type-2 receptor (Tgfbr2) expressing progenitor cell that has been shown in-vivo and in-vitro to have anatomical, ontogenic and slow-cycling expression profiles of progenitor joint cells [7, 8]. Ablation of the Tgfbr2 gene induces loss of tendon/ligament formation [9, 10]. Further research has shown that TGF-β-type-2(Tgfbr2) singling plays an essential role for tendon morphogenesis via regulating scleraxis, which is expressed in all the cells of tendon tissues as a key transcription factor for tenogenic differentiation [11]. Tgfbr2 expressing progenitor cells are maintained in postnatal tendon-bone junctions [8]. Furthermore, when treated with TGFβ, Tgfbr2 expressing progenitor cells, isolated from the early limb embryonic developing stage at E13.5-E14.5, express more tendon/ligament markers, including scleraxis and tenomodulin, suggesting that Tgfbr2 expressing cells may function as tendon/ligament progenitor cells at the early stage of the tendon/ligament morphogenesis [8]. By applying these unique Tgfbr2expressing progenitor cells to a multiphase tissue engineering scaffold that contains a continuous gradient between two different but contiguous structures, one can mimic the architecture, porosity, mechanical and immunochemical properties of a tendon-bone junction. It is anticipated that this novel approach to bone-tendon interfacial tissue engineering will avoid the use of pluripotent stem cells or the need to co-culture two or more different cell lines [3].

The tissue engineering scaffolds must be biocompatible, highly porous and biodegradable. They should also promote cell attachment, proliferation and differentiation and recruit fibroblasts that can secrete their own extracellular matrix resulting in the generation of living tissue. Various biomaterials have been used to develop scaffolds for tendon bone junction regeneration. For example, Yokoya et al., [12] compared three synthetic polymers: polytetrafluoroethylene (PTFE), poly-L-lactate-epsilon-caprolactone (PLC) and polyglycolic acid (PGA) for the repair of a tendon bone junction. It was found that the use of a polyglycolic acid (PGA) sheet promoted faster regeneration than the other two polymers. The PTFE sheet caused a chronic foreign body inflammatory response and PLC degrades very slowly so it is not considered a suitable scaffold material for tendon bone junction regeneration. The major disadvantages of using a PGA sheet is that its mechanical properties are marginally inferior to those of natural tendons but its use has significant potential for the regeneration of multiple tissue junctions.

In another example, Hong Wei et al., [13] developed a knitted poly-lactide-co-glycolide (PLGA) scaffold to repair the defect in an injured Achilles tendon. Bone marrow stromal cells (bMSCs) were seeded onto the scaffolds which were implanted in rabbits. The tissue formed by this scaffold was composed mainly of Type I and Type II collagen fibers and their strength was similar to that of natural tendon tissue after 12 weeks.

In this study, we are proposing to use braiding technology, which intertwines or braids several yarns together into a tubular structure to develop multiphase tissue engineering scaffold [14]. For many years this technology has been used to manufacture ropes, cords and shoe laces, and now it is being used in other fields such as medical textiles. Some of the applications of braiding technology in medical textiles includes development of sutures, stents, vascular grafts, nerve regeneration conduits and tissue engineering scaffolds for ligament, tendon, cartilage and liver tissues [15, 16].

The ultimate goal of this approach was to design a unique multiphase scaffold braided from resorbable poly(lactic acid) (PLA) yarns. Poly(lactic) acid fibers are thermoplastic, biocompatible and biodegradable. They have the potential to be used in wide range of applications in various fields such as healthcare, medicine, apparel, sportswear, furnishing, filtration, packaging and composites. In the medical device industry, it is used for making sutures, for surgical implants such as hernia repair meshes, bone plates, for tissue engineering scaffolds as well as bandages and wound dressings. Since the primary degradation product of Poly (lactic) acid is lactic acid, which is biocompatible and a normal product of healthy muscle function, it is widely used in a wide range of medical applications. The United States Food and Drug Administration (FDA) has approved the use of Poly (lactic) acid as a polymer material for human clinical use in a number of implantable enduses [17].

The first step reported here has been to fabricate two prototype scaffolds whose mechanical properties mimic those of soft tissue tendon and a third scaffold that mechanically mimics hard bone tissue. In addition, there was a need to demonstrate that the variation in their structure, porosity and mechanical properties, such as ultimate tensile strength and Young’s modulus were similar to both soft flexible tendon and hard bone tissues. These mechanical properties for natural tissues and for the braided scaffolds are described in the results section below. Furthermore, it was important to determine that the scaffolds promote the attachment, viability and proliferation of murine Tgfbr2-expressing joint progenitor cells.

## Methods

**Fig. 1:**
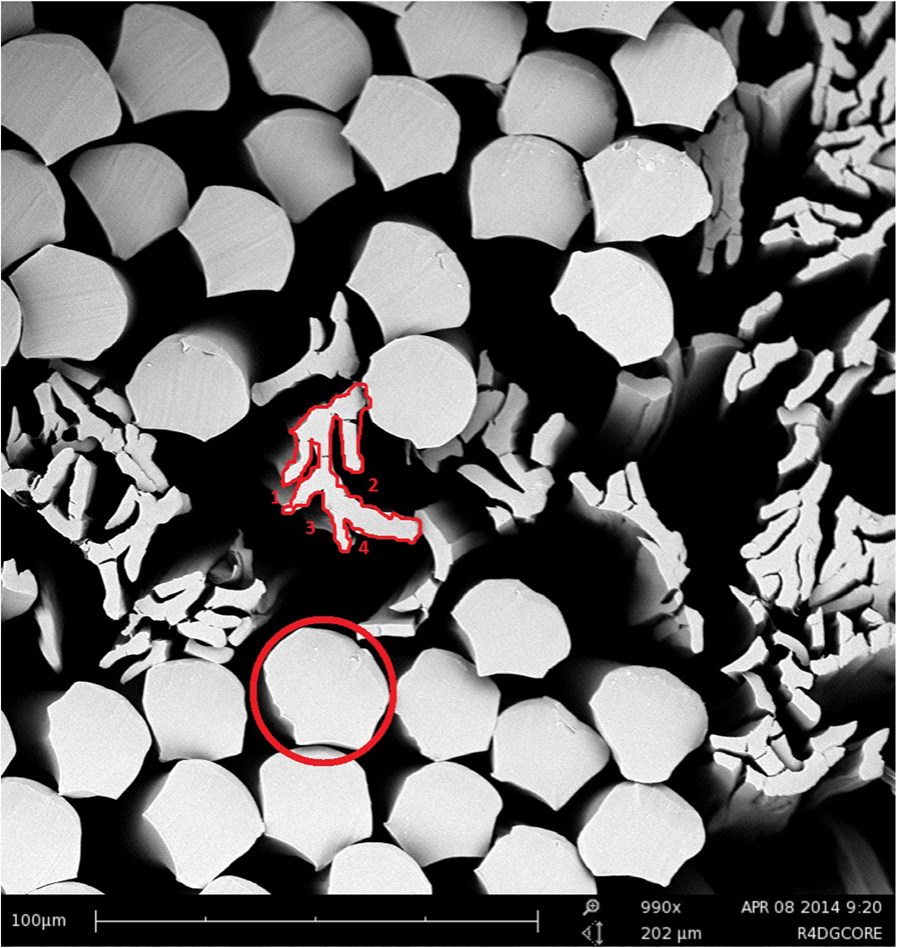
SEM image showing the cross-sectional shape of the 4DG fibers and round fibers

Two different types of specially designed multifilament poly(lactic acid) yarns were included in this study. Round fibers with a diameter of 25 μm and 4DG fibers having a cross-section with four deep grooves (4DG) with a thickness dimension of 4.5 μm (Fig. 1) were used to prepare three different scaffolds. They were spun and drawn at Fiber Innovation Technologies Inc. (Johnson City, TN) using a blended copolymer of > 98% poly(L-lactic acid) and < 2% poly(D-lactic acid) supplied by NatureWorks LLC (Minnetonka, MN). The yarn with the round fiber is 72 nominal denier per ply and the yarn with the 4DG fiber is 60 nominal denier per ply. Both the yarns are multifilament yarns with 18 filaments per ply. The basic properties of both fibers are listed in Table 1. The grooved 4DG fiber is a fiber with four deep grooves along the fiber whose surface area is three times larger than the traditional round fiber. The use of this novel experimental yarn with a much larger surface area was to evaluate its biological response to cell attachment, proliferation, and alignment.

**Table 1:** Basic properties of the fibers

Type of fiber	Fineness (den/filament)	Cross sectional shape	Surface area (circumference of cross section)	Major channel area (width*length)
Poly(lactic acid) 4DG	3	Grooved	375 μm	18.75 μm * 26 μm
Poly(lactic acid) round	4	Round	112.5 μm	NA

In order to mimic bone tissue, Scaffold No. 3 contains an additional core component that was inserted into the central lumen. The core component was a plied yarn. Since we were braiding a tubular scaffold with diameter of 1–1.5 mm, we decided to insert a 10 ply 60d/18f 4DG yarn so as to achieve the desired yarn density and porosity. To prepare a 10-ply core yarn, ten 4 DG 60d/18f poly(l-lactic acid) (PLA) multifilament yarns were twisted together on a Direct-twist-2A twister (Agteks, Ltd) at 150 rpm. This plied yarn served as the core yarn to braid Scaffold No. 3 which consist of poly(lactic acid) bilayer tubes braided from round fibers with a 10 ply grooved fiber central core insertion. Since the single 4DG yarns had insufficient strength to withstand the tension during the braiding process, all the 4DG yarns used to braid the walls of the scaffold were 3 ply yarns. The 3 ply yarns were prepared by the same machine that was used to ply the 10-ply core 4DG yarns. The yarn that was produced by Fiber Innovation Technology Inc. (Johnson City, TN, USA) was an undrawn round 170d/18f poly(l-lactic acid) (PLA) partially oriented yarn (POY) which was converted into a fully drawn 117d/18f poly(lactic acid) yarn (FOY) by using Model SW3 Drawing Tower (Hills Inc., Melbourne, FL) at the College of Textiles. Drawing was a two-step process at 76with a wind up speed of 380 m/min. The draw ratio was 1.9 to 1. The basic properties of the fully drawn round poly(lactic acid) yarns that were used to braid the scaffolds are shown in Table 2.

**Table 2:** Properties of fully drawn round poly(lactic acid) yarn

Drawn 117/18 poly(lactic acid)	Cross-section	Crystallinity (%)	Tg (^°^*C*)	Tm (^°^*C*)	Density (g/cm^3^)	Max Load (lbf)	Elongation at break (%)
	Round	31	60–65	173–178	1.24	0.721	23.34

All three types of scaffolds were braided using a Steeger USA 16-spindle braiding machine (Model K80/16–2008-SE) in this study. The braiding angle is calculated from the following equation [18]

$$ \theta ={\tan}^{-1}\frac{2\uppi \left(\mathrm{D}+2\mathrm{d}\right)\mathrm{P}}{\mathrm{C}} $$

where D is the diameter of the braided tube (inch), d is the diameter of the yarn (inch). P is the pick count (picks/inch) and C is the number of carriers. The average diameter of the braided tubes was first determined using a compression tester, but was also estimated by analyzing the images taken by SEM. The three scaffolds were:

- Scaffold 1: Poly(lactic acid) single wall hollow tube using round fibers. It is labeled RNC (Round No Core) in Table 3. This was designed to mimic the tendon region. Braiding technology was used to provide a flexible yet compression resistant scaffold that would maintain its dimensional stability after heat setting in a liquid culture media.
- Scaffold 2: Poly(lactic acid) single wall hollow tube using grooved and round fibers. This is labeled 4DGRNC (4DG Round No Core) in Table 3. This was also designed to mimic the tendon region, but by decreasing the pore size distribution in the wall of the scaffold it was anticipated this would prevent the leakage of cells into the hollow lumen.
- Scaffold 3: Poly(lactic acid) bilayer tube with an inserted knitted central core of both round and grooved fibers, referred to as 4DGRC (4DG Core) in Table 3. This was designed to mimic the bone region. The insertion of core fibers inside the hollow braided structure was designed to improve the stiffness, dimensional stability and mechanical strength of the scaffold so it would mimic natural bone. The morphology, porosity, tensile properties and biological performance of all three scaffolds were measured by the following experimental methods.

**Table 3:** Basic physical properties of the three types of braided scaffolds

Name and number of scaffold	Mass per unit length (mg)	Length (mm)	Diameter (mm)	Total porosity (%)	Pore size range (μm)	Braiding angle (^°^)
1: RNC	3.90	10	1.2	72.2	5–25	26
2: 4DGRNC	3.45	10	1.2	75.4	5–25	26
3: 4DGRC	5.65	10	1.2	60.4	5–25	26

### Morphology by scanning Electron microscopy (SEM)

The surface morphology and cross-sectional views of the three different scaffolds were observed using a Phenom G1 scanning electron microscope (Phenom, Netherlands) after sputter coating with gold-palladium in a SC7620 mini sputter coater (Quorum Technologies Inc., Canada). Images of the surface and cross-sectional views were captured at magnifications in the × 400 to × 1000 range.

### Total porosity and pore size

The total porosity of the scaffolds was calculated from the following equation [19]:

$$ \mathrm{Total}\ \mathrm{Porosity}\ \left(\%\right)=\left(1-{\mathrm{d}}_{\mathrm{S}}/{\mathrm{d}}_{\mathrm{PLA}}\right)\ \mathrm{x}\ 100 $$

Where,

d_S_ = the density of the braided scaffold

d_PLA_ = the density of the poly(lactic acid) polymer which is 1.24 g/cm^3^ [20].

The density of the braided poly(lactic acid) scaffolds was calculated from the mass of the scaffold and the cross sectional area of a 1 cm long section with a diameter range from 1.0 to 1.5 mm. The values for mass were measured experimentally to 4 decimal places using a Mettler H80 scientific balance.

Scanning electron microscopy was used to determine the average individual pore size and the pore size distribution of the three braided scaffolds since the size of the pores lay in the range of 0.01 μm – 10 μm. At least 10 specimens were visualized and measured using Image J software and the average values were calculated.

### Mechanical properties

The ultimate tensile strength of the three types of scaffolds was measured in the axial direction on an Instron mechanical tester following ASTM D5035–11 Standard Test Method for Breaking Force and Elongation of Textile Fabrics [21]. Five specimens for each sample were cut to a length of 40 mm and clamped between the jaws so as to provide a gauge length of 10 mm. The crosshead moved at a speed of 12 mm/min until the specimen failed. The maximum tensile strength was calculated from the measured value of maximum load. Young’s modulus, E, was determined from the initial linear portion on the slope of the stress/strain curve using the following equation:

$$ E=\frac{Tensile\ stress}{Tensile\ strain}=\frac{\sigma }{\varepsilon }=\frac{\raisebox{1ex}{$F$}\!\left/ \!\raisebox{-1ex}{$A$}\right.}{\raisebox{1ex}{$\Delta L$}\!\left/ \!\raisebox{-1ex}{$L$}\right.}=\frac{F\times L}{A\times \Delta L} $$

Where,

E = Young’s modulus (MPa)

F = Absolute force applied to the fabric (N)

A = Original cross-sectional area of the scaffold (mm^2^)

= Extension of the scaffold in the axial direction (mm)

L = Original gauge length (mm).

### In vitro cell culture study

#### Sample preparation

The three braided scaffolds were cut into 5 mm lengths and placed in a 96 well plate with one scaffold in each well. The scaffolds were sterilized using ethylene oxide in an Auprolene Model AN74ix sterilizer (Anderson Products, Inc.) for 12 h at ambient temperature. In order to coat the scaffold with serum, the scaffolds were immersed in 10% fetal bovine serum (FBS) and kept overnight in an incubator at 37 and 5% CO_2_.

#### Tgfbr2 expressing cells isolation and seeding

The Tgfbr2expressing cells were isolated from 13.5/14.5 day old embryos of tenogenic Tgfbr2-β-Gal-GFP-BAC mice as previously described [8]. The embryos were removed and separated from the pregnant female mice. The regions where the forelimbs and hindlimbs were developing were removed by viewing the embryos under a dissecting microscope. The tissues were cut into small pieces and shaken in Dispase (1u/ml) for up to 1 h digestion at 37. The cells were filtered through a pre-wetted 40 μm cell strainer to remove any clumps and then they were spun for 5 min at 1500 rpm. The PBS was carefully removed from the cell suspension which was re-suspended with about 1 ml micromass medium. The cell suspension was filtered again through a pre-wetted 40 μm cell strainer, and the cells were counted. The cells were then diluted with the micromass medium and taken for sorting.

After sorting, the GFP+ and GFP- cells were collected and counted. In line with previous experience of sorting Tgfbr2expressing cells, a total of 34.2 × 10 ^6^ cells generated only 229,827 GFP+ cells, which was a yield of 0.67% [8]. The pre-sorted and sorted cells with a total of 0.1–1.0 × 10^5^ in 10 μl micromass medium were seeded in the center of each prepared poly(lactic) acid scaffold which was then kept in the incubator at 37 and 5% CO_2_ for 1 h. Then 1 ml of micromass medium was pipetted into each well and changed every other day. The plates were incubated at 37 and 5% CO_2._ Three specimens were used for each sample together with a control which had only cells, no scaffold specimen.

The biological performance of the three scaffolds was evaluated at different time points by cell culture using Alamar Blue assay and laser scanning confocal microscopy (LSCM) with a live/dead stain.

#### AlamarBlue™ assay

The alamarBlue™ assay was used to evaluate cell viability and cell proliferation on the three different scaffolds at three different time points, namely: Day 3, 7 and 14. Living cells maintain a reducing environment inside the cytosol and the alamarBlue™ reagent uses this reducing power of the cells to confirm the viability. The alamarBlue™ reagent consists of an active component resazurin, which is a non-toxic, cell permeable compound that is blue in color and virtually non-fluorescent. When this compound enters a living cell, it is reduced into resorufin, a red colored compound which is highly fluorescent. This is the mechanism that the alamarBlue™ assay uses to quantify the viability of cells [22].

At each time point, the three different scaffolds were taken from the 96-well plate and transferred into a new plate. Then the medium from the old plate was pipetted out for the alamarBlue™ assay, which measured the level of fluorescence at the excitation wavelength range of 540 nm–570 nm on a Synergy micro-plate reader based on the alamarBlue™ assay kit (Life Technologies).

#### Laser scanning confocal microscope (LSCM) using live/dead stain

The migration and attachment of cells along the surface and within the internal structure of the three different scaffolds was observed by laser scanning confocal microscopy (LSCM) after 3 and 7 days of culture. A live/dead cell double staining kit (Sigma–Aldrich) was used to visualize and differentiate between the live and dead cells using a Zeiss LSM 710 laser scanning confocal microscope (LSCM) (Carl Zeiss Micro imaging, USA). The staining kit consisted of two components, namely component A- Calcein-AM and component B-Ethidium homodimer-1 (EthD-1) solutions to stain live and dead cells respectively. Calcein AM is able to penetrate inside live cells, reacts with esterase and changes into calcein, which produces an intense green fluorescence, while Ethidium homodimer-1 enters dead cells and reacts with the damaged membrane to produce bright red fluorescence. The difference in wavelength of the two components in the staining kit enabled us to distinguish between the live and dead cells. If the cells were alive, they appeared green under the confocal microscope, and if the cells were dead they appeared red. The wavelengths used for imaging the live cells were λex~ 494 nm and λem~ 517 nm, whereas the wavelengths used to view the dead cells were λex~ 528 nm and λem~ 617 nm based on the kit. Three-dimensional image reconstruction and analysis were performed using ZEN software (Carl Zeiss Micro imaging, USA).

### Statistical analysis

Descriptive statistics were used to calculate the mean and standard deviation for the experimental data measured on each sample. The standard deviation was used to generate the error bars in the figures and a two-tailed t-test was carried out to confirm significant differences between two mean values at a 95% confidence interval (*p* > 0.05).

## Results

### Characterization of the braided scaffold structure

**Fig. 2:**
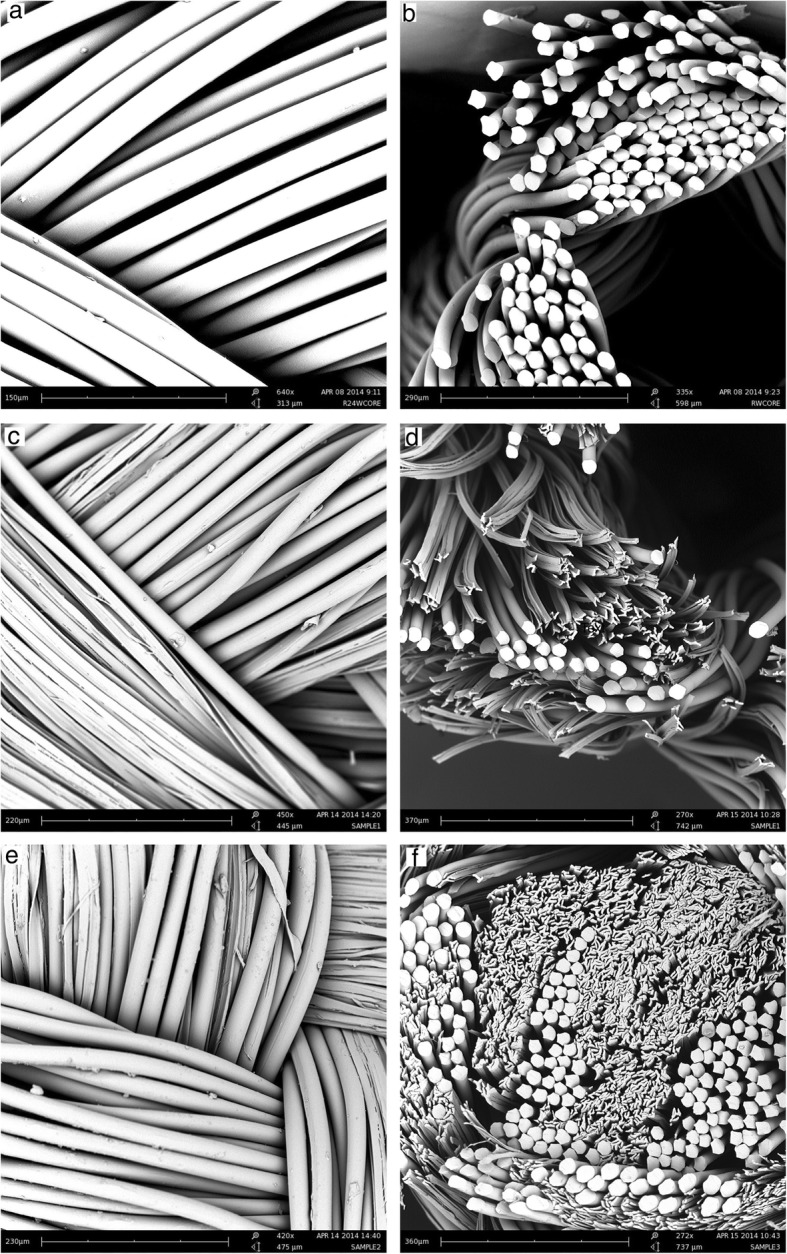
Surface & cross-sectional views of Scaffold 1 - hollow tube braided from round fibers (**a-b**), Scaffold 2 - hollow tube braided from a mixture of 4DG and round fibers (**c-d**), Scaffold 3 - concentric bilayer tube with a mixture of 4DG and round fibers in the central lumen (**e-f**)

Microstructural images of the scaffolds’ cross-section and surfaces were taken under scanning electron microscopy at different magnifications. Figure 2a-b shows the cross sectional (a) and longitudinal (b) surface views of the round hollow scaffolds with no central core (Scaffold 1). It shows the smooth surface of the round poly(lactic acid) fibers. Figure 2c-d shows the cross sectional (c) and rougher longitudinal (d) surface views of the mixture of 4DG and round fibers (Scaffold 2), which have a larger surface area compared to Scaffold 1. Figure 2e-f shows the cross sectional (e) and longitudinal (f) views of the poly(lactic acid) concentric bilayer tube with a mixture of round and 4DG fibers inserted in the central core (Scaffold 3).

### Physical properties of the braided scaffolds

Braids have several advantages over other types of tubular structures. They are soft, flexible and semipermeable tubes that can be placed inside living organisms using a trocar or a catheter without major complications.

**Fig. 3:**
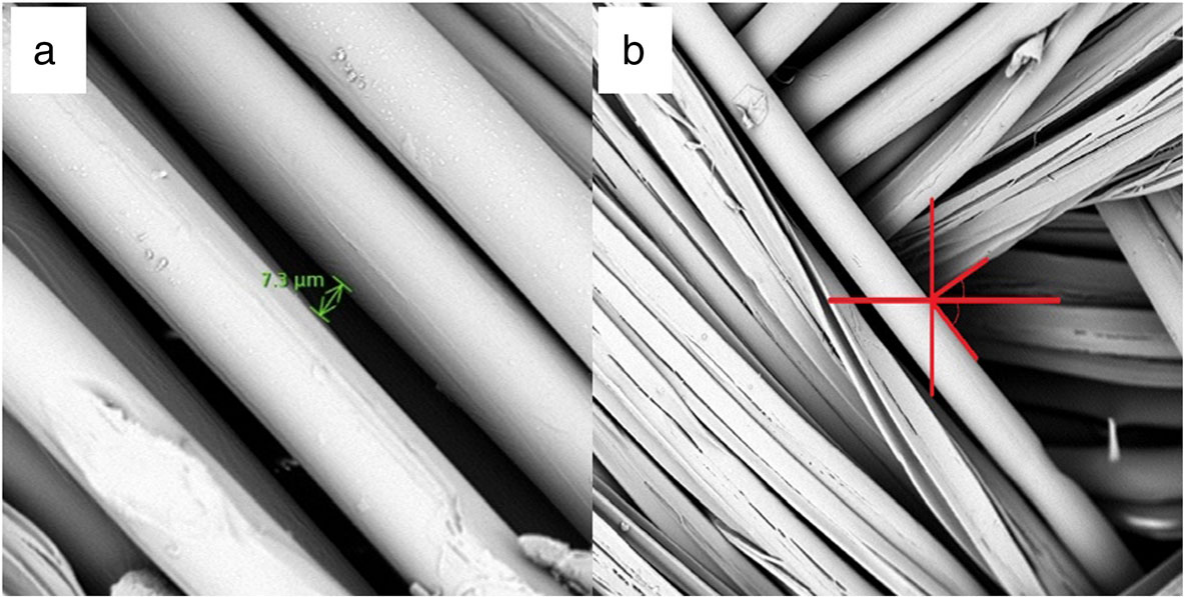
SEM images of the poly(lactic acid) braided scaffolds with pore size (**a**) and braiding angle measurement (**b**)

All three braids had the same diameter (1.2 mm), the same pick count (24 picks/inch) and the same number of carriers (16) as they were all braided on the same Steeger braiding machine. As reported earlier, the average diameter of the round fibers was 25 μm and the average thickness of the 4DG fibers was 4.5 μm. By using these measurements in the braiding angle equation, the calculated braiding angle was found to be 26, which was in agreement with the value measured from SEM images showed in Fig. 3b. The average pore size where the braided yarns crossed was close to zero, whereas between individual filaments the pore size ranged from 5 to 25 μm (Fig. 3a). These pores contributed to the exchange of oxygen and provided nutrition. The basic properties of the three types of braided scaffolds developed for this study are summarized in Table 3.

### Mechanical properties

**Fig. 4:**
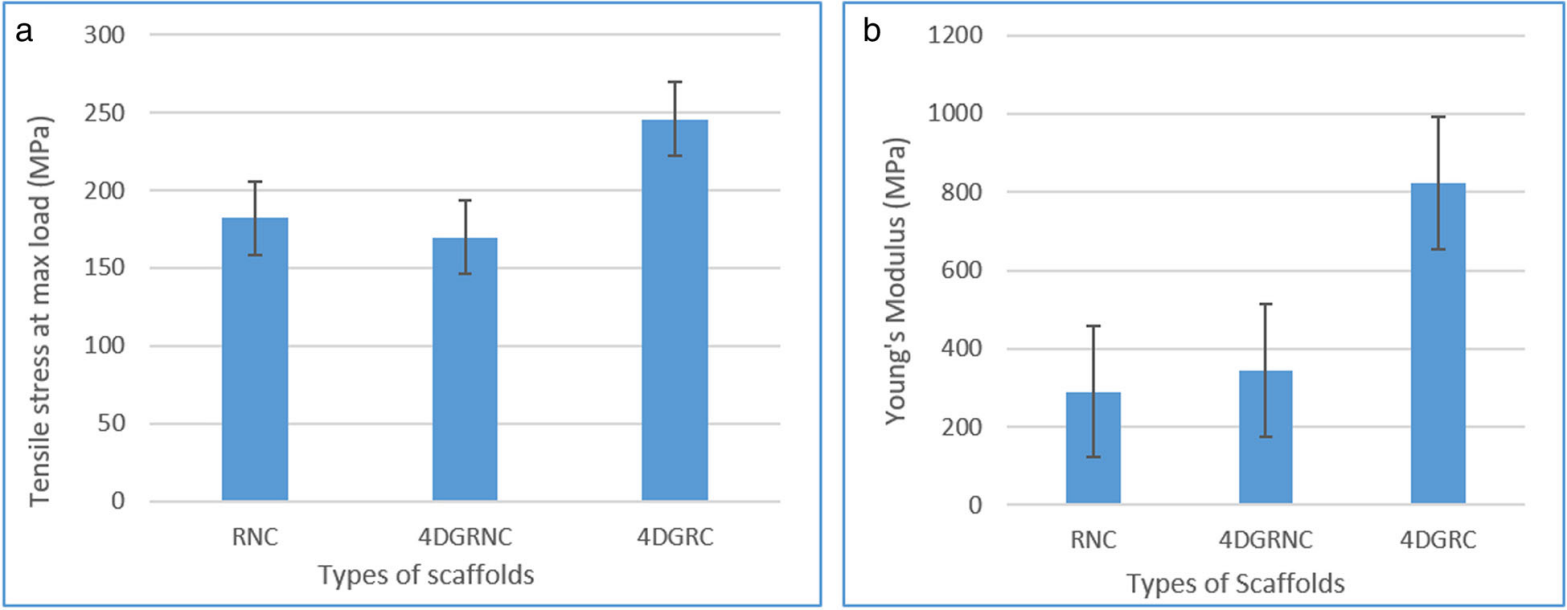
Ultimate tensile strength (**a**) and Young’s modulus values (**b**) of three types of scaffolds: Scaffold 1 and Scaffold 2 were hollow scaffolds, Scaffold 3 contained a core

The three fabricated scaffolds were evaluated to ensure that they had sufficient overall strength and integrity to function as a load-bearing tendon/bone tissue junction, and also to mimic the elastic Young’s modulus of the separate tendon and bone components. Figure 4a shows that the ultimate tensile strength of all three scaffolds were in comparison to the ultimate strength of human bone (700–18,000 MPa) and human tendon (250 MPa) [23]. The tensile strength of the Scaffold 3 scaffold, which was designed to mimic bone, was significantly higher than for the two hollow scaffolds without a core, which mimicked the tendon. The ultimate tensile strengths of these two hollow scaffolds were not significantly different from each other (*p* value = 0.07 ≥ 0.05). Hence the insertion of a core within the braided structure increased the tensile strength sufficiently to mimic hard bone tissue.

Figure 4b shows the Young’s modulus values for all three scaffolds. The value for the bilayer tube with the central core was significantly higher than for the other two hollow scaffolds (*p* ≤ 0.05), between which there was no significant difference (*p* ≥ 0.05). By comparing these Young’s modulus values with those for human tendon and bone in Table 4, it can be seen that the two hollow scaffolds mimic the properties of the human tendon [23], whereas the bilayer braided scaffold with core insertion falls within the range of Young’s modulus for human bone [23]. Thus, the three different braided scaffold structures provide a range of mechanical properties that mimic the component parts of a human tendon/bone tissue junction.

**Table 4:** Comparison of Young’s Modulus of the three braided scaffolds with human natural tissues

Young’sModulus (MPa)	Poly(lactic acid) hollow tube with round fibers (RNC)	Poly(lactic acid) hollow tube with 4DG & round fibers (4DGRNC)	Poly(lactic acid) bilayer sheath with 4DG core insertion (4DGRC)	Human Tendon	Human Bone
	290	342	822	250	700–18,000

### Biological performance of the scaffolds

#### Cell viability and proliferation

The alamarBlue™ assay was used to evaluate the extent of cell viability and cell proliferation of the Tgfbr2 expressing cells on the three different types of scaffolds measured at Day 3, 7 and 14.

**Fig. 5:**
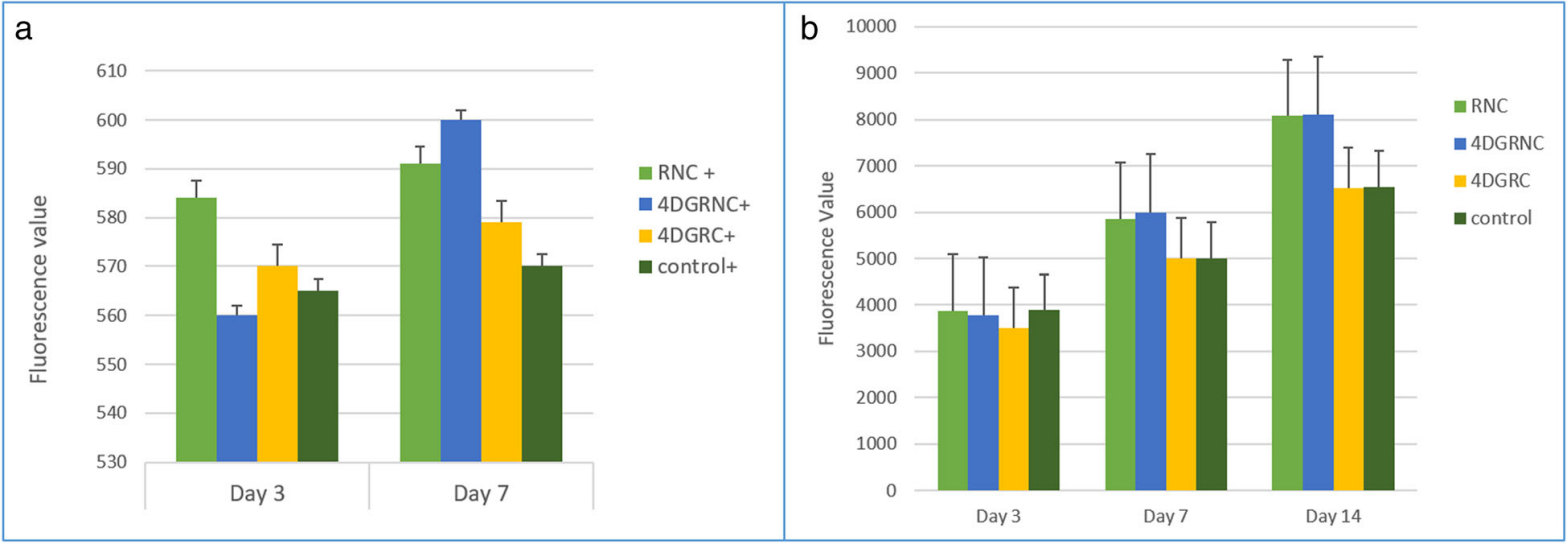
Fluorescence values of the Tgfbr2 positive cells (**a**) at Day 3 and Day 7 and presorted cells (**b**) at Day 3, 7 and 14

Figure 5a shows the fluorescence values of the Tgfbr2 positive cells measured on Day 3 and Day 7, whereas Fig. 5b shows the fluorescence values of the presorted cells at Day 3, 7 and 14. On comparing the extent of cell proliferation at different time points for the presorted cells, it can be seen that on Day 14 the cell proliferation was significantly higher than on Day 3 (*p* ≤ 0.05). This shows that the cells were continuously proliferating and indicates that all three types of scaffolds were biocompatible and non-cytotoxic. By comparing the fluorescence values for the scaffolds and the well plate controls, it can be concluded that there was greater cell viability on the scaffolds compared to the plates. This may have been due to the scaffolds being coated with fetal bovine serum before seeding, whereas the well plates were uncoated. The fluorescence values of the Tgfbr2 positive cells (Fig. 5a) were much lower than for the presorted cells (Fig. 5b), due primarily to the smaller seeding density of the positive cells.

On comparing the three different scaffolds (Fig. 5b) on Day 3, the hollow scaffold (Scaffold 1) with round fibers showed slightly higher viability and proliferation compared to the other hollow scaffold (Scaffold 2) and the bilayer scaffold with the central core (Scaffold 3). However, on Day 7 and Day 14 the two hollow scaffolds gave similar results, although the 4DG fibers had a marginally faster rate of proliferation, which may have been due to the 4DG grooved fibers.

The bilayer scaffold with the central core (Scaffold 3) gave a marginally slower rate of cell proliferation compared with the hollow scaffolds, which is thought to be due to its limited porosity. In summary, the hollow scaffolds (Scaffold 1) coated with fetal bovine serum showed the highest cell viability and cell proliferation among the three different braided scaffolds.

#### Cell attachment and cell infiltration

**Fig. 6:**
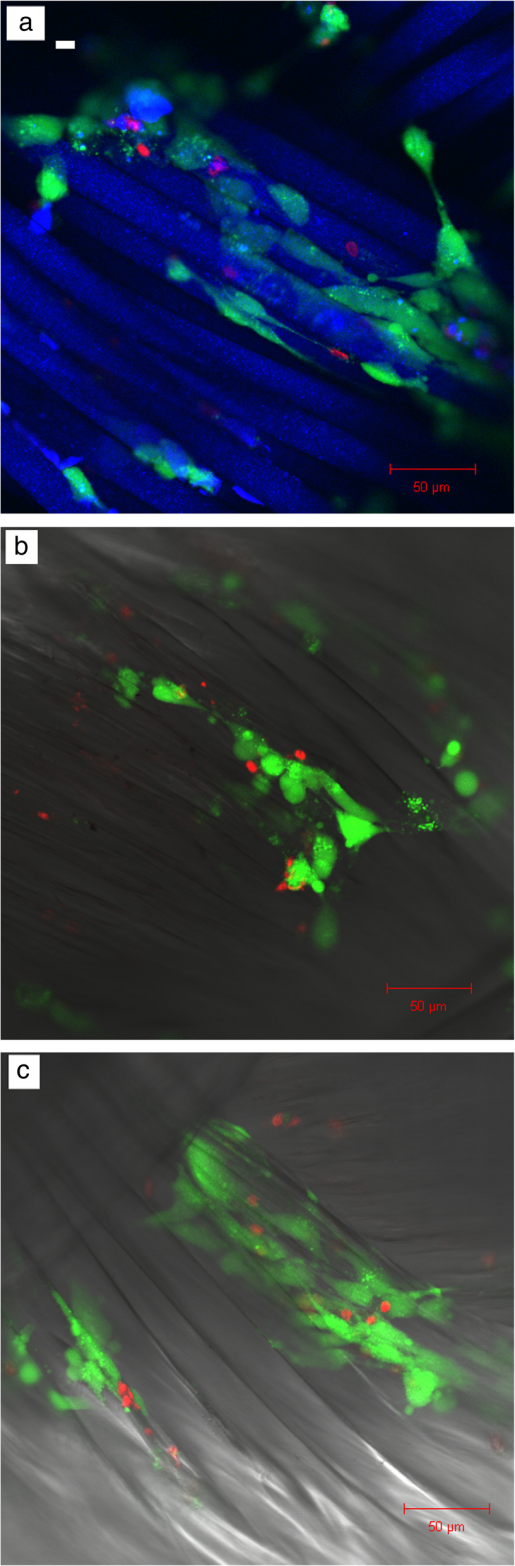
Cell attachment on the three different braided scaffolds **a** Scaffold 1; **b** Scaffold 2; **c** Scaffold 3 on Day 7 showing live (green) and dead (red) cells on the same images

Laser scanning confocal microscopy was used to determine the extent of infiltration and attachment of the cells on the three different scaffolds. In addition, the viability of the cells was determined using a live/dead assay (Sigma-Aldrich). The images of all three scaffolds with cells taken on Day 7 are shown in Fig. 6 at a lower magnification. The images indicate that the cells penetrated inside the scaffolds and attached themselves to the poly(lactic acid) fibers, and the ratio of green live cells to red dead cells was greater than 1. In order to obtain a clearer view of the cellular performance, images containing only live cells and only dead cells were obtained separately using the ZEN software.

**Fig. 7:**
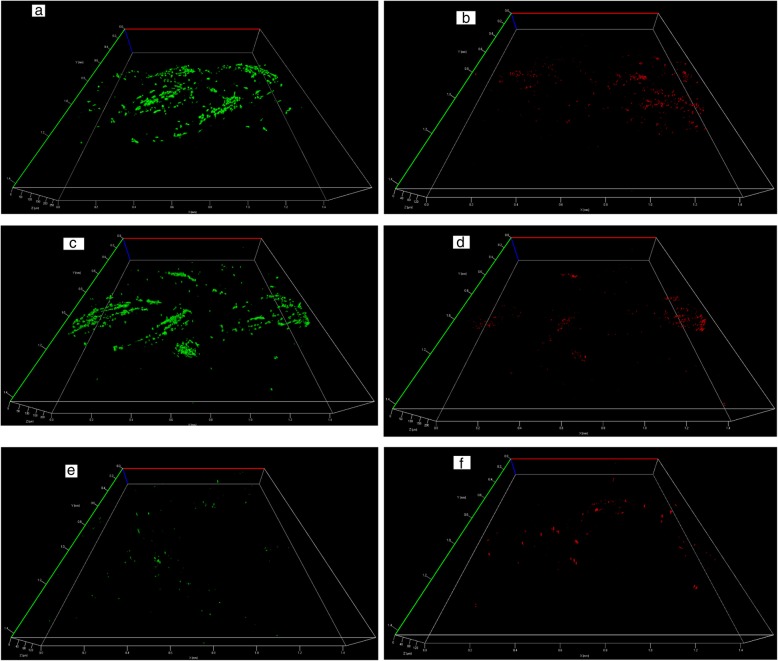
Three dimensional images of live (green) and dead (red) cells on Day 3: **a-b** Scaffold 1- hollow scaffold with round fibers, **c-d** Scaffold 2 - hollow scaffold with 4DG fibers and **e-f** Scaffold 3 - bilayer scaffold with a central core on Day 3

Figures 7 show the three-dimensional images of live and dead cells at Day 3 on the hollow scaffold with round fibers (a-b), on the hollow scaffold with 4DG fibers (c-d) and on the bilayer scaffold with a central core (e-f) respectively. At Day 3 the cell viability was the highest for the hollow scaffold with round fibers (Scaffold 1), followed by the hollow scaffold with 4DG fibers (Scaffold 2). This was in agreement with the Alamar blue results.

**Fig. 8:**
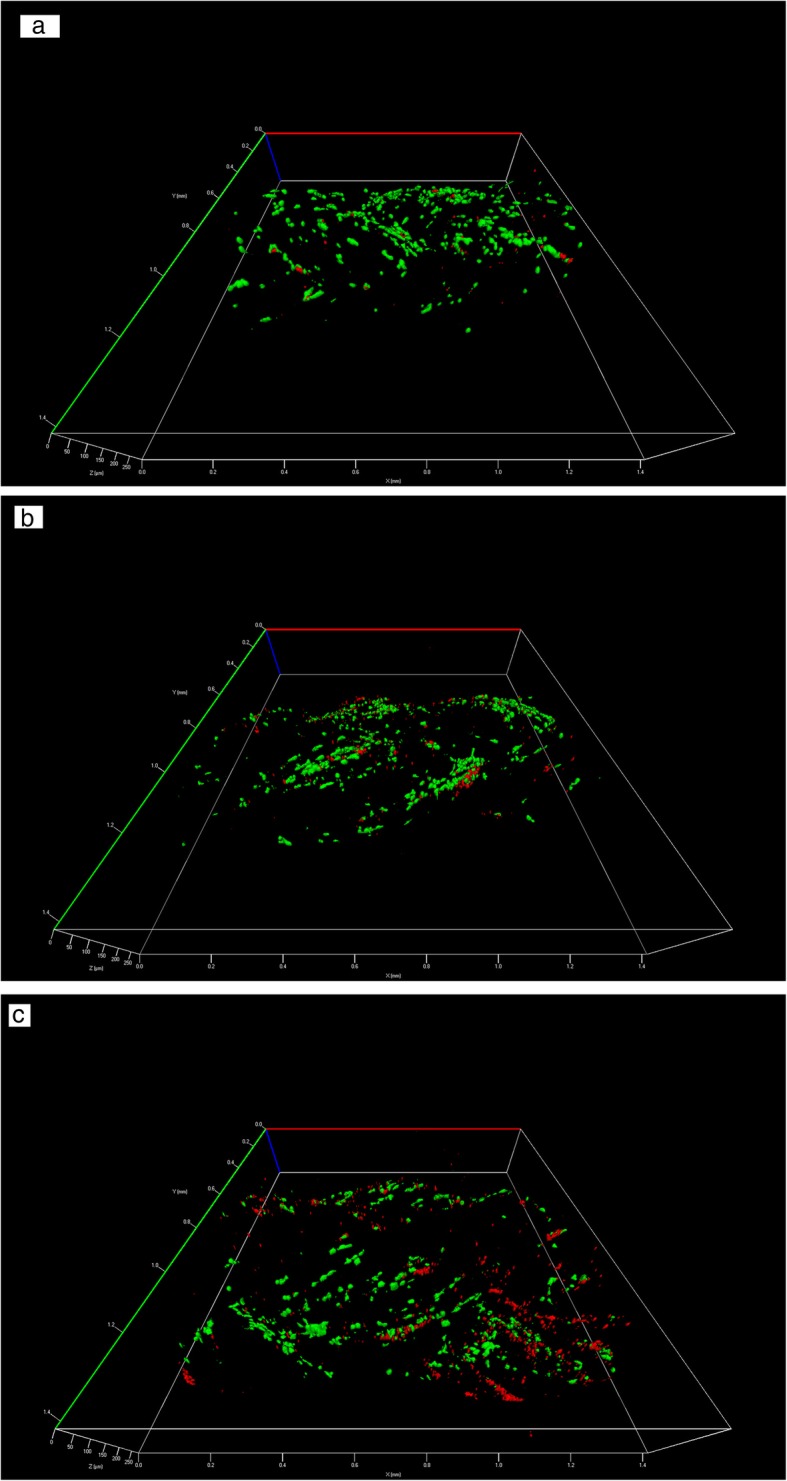
Three-dimensional image of combined live (green) and dead (red) cells on Day 7: **a** Scaffold 1 - hollow scaffold with round fibers, **b** Scaffold 2 - hollow scaffold with 4DG fibers and, **c** Scaffold 3 - bilayer scaffold with a central core

Figures 8 show the three-dimensional combined LSCM images of live and dead cells on the three scaffolds at Day 7. Compared to Day 3, the images show more green cells, confirming that cells were continuously proliferating, and that the poly(lactic acid) fibers supported cell viability even after 7 days of culture. The cells were observed to be present throughout the thickness of the scaffold, which confirms that the poly(lactic acid) fibers were biocompatible, and that the experimentally braided prototype structures were able to promote cell viability, proliferation and infiltration. Compared to Day 3, the confocal images of the multilayer scaffold with the central core showed more green cells, indicating that cell infiltration was enhanced by including a central core within the braided structure to serve as a guidance component.

## Discussion

The surface morphology of all three braided structures as seen from the scanning electron microscopy images exhibits a porous structure due to the interlocking of the braided yarns. The ideal tissue engineered scaffold should have a total porosity in the range of 50–80% to support the culture of cells and the diffusion of nutrients throughout the whole structure [24]. The individual pore size should be in the range of 5–15 μm for fibroblast ingrowth and around 200 μm for osteo-conduction [24]. As seen in Table 1, the total porosity of all three prototype scaffolds was within the required range, indicating that the braided scaffold structures should be able to support cell ingrowth, uniform cell distribution and the transfer of oxygen and nutrients. While the pore size for the two scaffolds, Scaffold 1 and Scaffold 2, that were mimicking the tendon lay in the ideal range for tenocyte ingrowth, the average pore size of the Scaffold 3 scaffold that was mimicking bone could have been larger in order to facilitate osteo-conduction. However, it was braided from 4DG fibers with deep grooves on the surface, which increased the surface area of the scaffold and improved the penetration of cells as seen in Fig. 8c.

In terms of the scaffold’s mechanical performance, the insertion of core fibers inside the central lumen improved its stiffness, dimensional stability and tensile strength compared to the hollow scaffolds. These data confirm that this multiphase structure has the ability to mimic the mechanical properties of natural bone. The heat setting treatment was successful in improving the rigidity of the scaffolds and maintaining their dimensional stability so as to avoid shrinkage during long term immersion in liquid culture media.

The incorporation of grooved poly(lactic acid) fibers marginally improved the biological properties. Although there was little difference in cell proliferation and penetration between the round and grooved 4DG fibers, the confocal microscope images showed that the murine Tgfbr2 expressing joint progenitor cells were attached and aligned within the grooves of the 4DG fibers as had been previously hypothesized.

Future work will focus on designing the scaffolds with increased pore size in order to promote cell migration and penetration into the scaffold. Tgfbr2 expressing joint progenitor cells will be co-cultured with poly(lactic acid) scaffolds to check if it still maintains its deferential ability. We will also focus on in vivo animal studies and clinical trials since they are necessary to evaluate the clinical capability of the specially designed scaffolds for regeneration of tendon/bone junction tissue.

## Conclusions

In this study, a series of specially designed biodegradable scaffolds for tendon-bone junction regeneration has been successfully fabricated from poly (lactic acid) (PLA) yarns using braiding technology. By planning the design of the interlocking braided yarns, the pore size distribution in the wall of the scaffold was small enough to prevent the cells from leaking into the central hollow space in the lumen. At the same time the porosity of the scaffold wall was large enough to facilitate cellular ingrowth and the transfer of oxygen and nutrients. The pore size of the hollow scaffolds mimicking the tendon was ideal for tenocyte ingrowth, whereas the average pore size of the scaffold with the additional central core component could have been larger in order to facilitate osteo-conduction. The three different types of scaffolds showed a wide range of mechanical properties that have the potential to be used as the scaffold for regeneration of a tendon bone junction.

Our study provided a great basis for further application of using a combination of our unique Tgfbr2 expressing joint progenitor cells with degradable scaffolds from poly(lactic acid) fibers for tendon-bone junction tissue engineering.

## Abbreviations

4DG: Four deep grooves
4DGRC: 4DG core
4DGRNC: 4DG round no core
FOY: Fully oriented yarn
ITE: Interfacial tissue engineering
PLA: Polylactic acid
POY: Partially oriented yarn
RNC: Round no core
Tgfbr2: Transforming growth factor beta type II receptor
